# Trait Impulsivity and Choice Impulsivity in Young Adult Students With Probable Binge Eating Disorder

**DOI:** 10.3389/fpsyt.2022.838700

**Published:** 2022-04-05

**Authors:** Wan-Sen Yan, Dan-Hui Zheng, Meng-Meng Liu

**Affiliations:** Department of Psychology, School of Medical Humanitarians, Guizhou Medical University, Guiyang, China

**Keywords:** binge eating disorder, impulsivity, discounting task, BIS-11, UPPS-P

## Abstract

**Background:**

Binge eating disorder (BED) as a public health problem has been included in the Diagnostic and Statistical Manual of Mental Disorders, Fifth Edition (DSM-5). Akin to addictive disorders, impulsivity-related neuropsychological constructs might be potentially involved in the onset and development of BED. However, it remains unclear which facets of impulsivity are connected to overeating and binge eating behaviors among non-clinical populations. The present study aimed to detect the relationship between impulsivity and binge eating both on the personality-trait and behavioral-choice levels in undiagnosed young adults.

**Methods:**

Fifty-eight individuals with probable BED and 59 healthy controls, matched on age, gender, and educational level, were assessed by using a series of self-report measurements, including the Barratt Impulsiveness Scale (BIS-11), UPPS-P Impulsive Behaviors Scale (UPPS-P), Delay Discounting Test (DDT), and Probability Discounting Test (PDT).

**Results:**

Multivariate analysis of variance models revealed that compared with healthy controls, the probable BED group showed elevated scores on the BIS-11 Attentional and Motor impulsiveness, and on the UPPS-P Negative Urgency, Positive Urgency, and Lack of Perseverance. However, the probable BED subjects had similar discounting rates on the DDT and PDT with healthy controls. Regression models found that Negative Urgency was the only positive predictor of binge eating behavior.

**Conclusions:**

These findings suggested that typical facets of trait impulsivity, which have been recognized in addictive disorders, were associated with binge eating in young adults, whereas choice impulsivity was not aberrantly seen in the same probable BED sample. This study might promote a better understanding of the pathogenesis of BED.

## Introduction

Binge eating disorder (BED) is characterized by overwhelming eating desire with recurrent episodes of binge eating (at least once a week during the last 3 months) and lack of control over binge-eating behavior (1). BED has been included as a separate category within the Feeding and Eating Disorders in the latest version of the Diagnostic and Statistical Manual of Mental Disorders (DSM-5) (2). The lifetime prevalence for BED in adults is about 2%, with women having a higher risk than men (3). The prevalence of BED among obese adolescents aged 12–17 years has been reported over 30% in recent literature (4). Generally, adolescents and young adults have a high risk for BED due to their immature cognitive control abilities (5, 6). Interestingly, although most studies focused on clinical samples of BED, some data showed that in non-clinical populations with a normal body mass index (BMI), over-eating behavior could also be seen with an increase in the risk of developing into BED in these young adults (7, 8). Nevertheless, it remains unclear which neuropsychological constructs might be potentially linked to BED among general adolescents and young adults.

Impulsivity is a hallmark feature in various mental disorders including addictive behaviors, as well as in so-called “food addiction”, which has been largely controversial (8). Many studies suggested that impulsivity might be a vulnerability trait for both behavioral and substance-related addictions (9–11). Importantly, individuals with BED and substance abusers shared similar intense cravings, disinhibition over the intake of foods or drugs, and altered reward sensitivity (12, 13). Therefore, impulsivity might also play a part in the processes of BED. However, the relationship between impulsivity and BED remains to be further understood.

Impulsivity refers to a tendency to act without careful thinking or to react prematurely (14, 15). Although impulsivity is a multifaceted construct, at least two different connotations of impulsivity may be separately detected with different measurements (16). Specifically, personality-level trait impulsivity is usually measured by self-report scales such as the Barratt Impulsiveness Scale (BIS-11) (17), while behavioral-level choice impulsivity is mostly assessed by reward discounting tasks such as the Delay-discounting Test (DDT) (18).

Trait impulsivity is a stable and inheritable feature with self-reported attributions of self-regulatory ability (19). Many previous studies have linked trait impulsivity to binge eating behaviors in clinical patients with BED (20, 21). Nevertheless, these samples always had high comorbidity with attention-deficit/hyperactivity disorder (ADHD) (22), anxiety disorders (23), and substance use disorders (24), which might lead to confounding results when detecting the relationship between impulsivity and BED. Moreover, limited data on the associations of trait impulsivity and BED have been incongruous among general populations. Some data suggested that heightened impulsivity was found in young adults with BED compared to healthy controls (25–28), while other studies showed no group differences (29, 30). Findings were also inconsistent when specific facets of impulsive traits were taken into account (31, 32). One prior study found that Attentional, Motor, and Non-planning Impulsiveness were significantly related to binge eating in normal-weighted women (27), while another study showed that only Attentional and Motor Impulsiveness were elevated in obese patients with overeating (30).

Comparatively, choice impulsivity is considered an irrational decision-making process influenced by motivations and affects (33, 34). Meta-analyses have demonstrated that increased choice impulsivity might be particularly relevant to BED (35–37). Clinical patients with BED, including both normal-weighted and over-weighted, displayed steeper delay discounting than healthy controls on the DDT (38, 39). Nonetheless, it is unclear whether this aberrant delay discounting was truly connected to BED itself or rooted in the comorbid psychiatric disorders in these clinical patients (40). Despite little evidence, several studies with non-clinical samples revealed that adults with BED exhibited steeper delay discounting compared to controls (41, 42). However, negative results also showed that BED individuals and healthy controls had no differences on delay discounting tasks (43, 44). Regarding probability discounting, limited studies suggested that obese women with BED tended to discount probabilistic rewards less steeply than healthy controls (45, 46), though our prior data displayed similar probability discounting in young adults with and without BED (47). Therefore, more studies are needed to elucidate the relationship between binge-eating behaviors and choice impulsivity in general populations.

The current study aimed to further detect the associations between impulsivity and binge eating among non-treatment-seeking samples. The Barratt Impulsiveness Scale-11 (BIS-11) and UPPS-P Impulsive Behaviors Scale (UPPS-P) were used to measure trait impulsivity, and the Delay Discounting test (DDT) and Probability Discounting test (PDT) were used to assess choice impulsivity, comparing probable BED subjects with healthy controls. It was generally hypothesized that heightened trait impulsivity and choice impulsivity would be linked to BED, as possible risk factors or vulnerability markers for binge eating behaviors.

## Methods

### Participants and Procedure

Participants were recruited through posters from a local university in Guiyang, China. Power analyses (48) were conducted to determine a target sample size (Cohen' s *d* = 0.4, α = 0.05, 1 – β = 0.8, F tests, G^*^Power), with a minimum sample size of 52 (at least *n* = 26 in each group). All subjects were invited to provide demographic information and complete a series of self-report questionnaires in the laboratory. Inclusion criteria included: (1) 18–25 years of age, and (2) willingness to participate in this study. Exclusion criteria included: (1) past or current severe psychiatric disorders (e.g., schizophrenia, bipolar disorder), (2) a history of illegal psychoactive substance use (e.g., cocaine, heroin, amphetamine), (3) brain trauma or neurological diseases, and (4) severe somatic diseases or special physical conditions that were inappropriate (e.g., menstrual period for women), all of which were evaluated by self-reports.

Probable BED status was estimated by using the Chinese version of the Binge Eating Scale (BES) (47), on which a total score of ≥18 indicates probable binge eating disorder (49). Finally, the probable BED group (pBED) consisted of 58 subjects (mean age = 19.34 ± 1.15 years; 10 men, 17.24%; mean BES score = 21.60 ± 3.29) according to the BES scores. The healthy controls (HCs) included 59 subjects, matched on age, gender, and educational level with the probable BED group (mean age = 19.12 ± 0.77 years; 10 men, 16.94%; mean BES score = 5.47 ± 2.22). All subjects gave informed consent and were compensated with a gift equal to RMB ¥50. The current study was reviewed and approved by the Human Research Ethics Committee at the Guizhou Medical University. The proposed study design, recruitment process, and our plans to compensate the participants were in accordance with the Declaration of Helsinki.

### Measures

#### Demographics

A brief self-report questionnaire was employed to collect demographic data of the subjects, including age, gender, ethnicity, and home locality. Standard procedures were used to measure weight and height, and then body mass index (BMI) was calculated as weight divided by the square of height (i.e., kg/m^2^). The participants also reported their smoking and drinking behaviors in the past 30 days on two questions (“Have you smoked at least one cigarette in the past 30 days?” and “Did you take at least one drink in the past 30 days?”).

#### Binge-Eating Behavior

The Binge Eating Scale (BES) (47, 50, 51), was used to screen binge eating behavior. The BES is a 16-item self-report questionnaire designed to assess behavioral, emotional, and cognitive symptoms of binge eating. Items were rated on a five-point Likert scale from 0 (not at all) to 4 (very much), with a total score ranging from 0 to 46. Higher total scores indicate more severe binge eating problems, with a score of ≥18 indicating probable binge eating disorder (BED) (49, 51). The Cronbach's α was 0.863 in this study.

#### Trait Impulsivity

Participants completed the Barratt Impulsiveness Scale (BIS-11) (17), a 30-item self-report inventory that measures impulsive personality in terms of three factors: Motor Impulsiveness, Attentional Impulsiveness, and Non-planning Impulsiveness. Items were rated on a four-point Likert scale. A higher score of each dimension indicates a higher level of trait impulsivity. The Cronbach's α for the BIS-11 was 0.796 in this study. Subjects also completed the UPPS-P Impulsive Behaviors Scale (UPPS-P) (15, 52), a 59-item self-report questionnaire used to assess five dimensions of impulsive personality: Sensation Seeking, Lack of Premeditation, Lack of Perseverance, Negative Urgency, and Positive Urgency. Items were rated on a four-point Likert scale. The Cronbach's α for the UPPS-P was 0.878.

#### Choice Impulsivity

The Delay Discounting Test (DDT) and Probability Discounting Test (PDT) were used to evaluate choice impulsivity. Both tasks were designed to evaluate discounting degrees of hypothetical monetary rewards. The DDT (18) is a fixed serial of a 27-item choice questionnaire between a smaller immediate monetary reward and a larger delayed monetary reward. For the DDT, *k* parameter indicates the degree of delay discounting, calculated by the equation: *V* = *A*/(1 + *kD*). In this equation, *V* refers to the individual subjective value of the delayed reward, *A* is the nominal amount of the delayed reward, and *D* is the length of the delay. A higher *k* indicates a higher degree of delay discounting. The PDT (53) is a three-part monetary choice questionnaire, with 10 items in each part. Participants were told to choose between a smaller amount of monetary reward obtained for sure and a larger amount of monetary reward obtained probabilistically (e.g., “$20 for sure” vs. “10% chance of obtaining $80”). The *h* parameter is calculated by the hyperbolic equation: *V* = *A*/(*1* + *h*θ). In this equation, *V* refers to the present subjective value of the probabilistic reward *A*. A lower *h* value implies that the probabilistic rewards are less steeply discounted, suggesting a reduction in risk aversion. The *k* and *h* values were log-transformed in analyses.

### Statistical Analyses

Data analysis was performed with the Statistical Package for the Social Sciences for Windows, Version 22.0 (SPSS Inc., Chicago, IL, USA). Chi-Square tests were used to test group differences on categorical variables (i.e., ethnicity, gender, home locality). *t*-Tests were adopted to analyze group differences on BMI and age. Multivariate analysis of variance (mANOVA) models were used to compare task scores between the two groups. Partial correlations were tested between the BIS-11, UPPS-P, DDT, PDT, and BES scores, controlling for age, BMI, gender, ethnicity, home locality, smoking, and drinking status. In addition, a multivariate linear regression analysis was conducted to test the effects of impulsivity measures on BES scores, and logistic regression analyses were tested for the predictive effects of impulsivity scores on binge eating behavior. According to the standardized variance inflation factor (*VIF*), multi-collinearity was not a problem for any variable in these regression models (*VIF* < 10). Significance was defined as *p* < 0.05, two-tailed.

## Results

### Demographic Characteristics

Table 1 illustrated the demographics and task scores of the two groups. The pBED group had a higher BMI than the HCs (*t* = 4.18, *p* = 0.001). No between-group differences were found for age (*p* = 0.214), ethnicity (*p* = 0.649), gender (*p* = 0.967), or home locality (*p* = 0.872).

**Table 1:** Demographic characteristics and impulsivity measures of the sample (*N* = 117).

**Variables**	**pBED group (*n* = 58)**	**HCs (*n* = 59)**	***χ^2^*/*t***	***p***
Age, years (M ± SD)	19.34 ± 1.15	19.12 ± 0.77	1.25	0.214
BMI, kg/m^2^ (M ± SD)	22.07 ± 3.53	20.06 ± 0.95	4.18	0.001
Ethnicity, Hans *n* (%)	31 (53.4)	34 (57.6)	0.21	0.649
Gender, male *n* (%)	10 (17.2)	10 (16.9)	0.01	0.967
Home locality, Urban *n* (%)	44 (75.9)	44 (74.6)	0.03	0.872
Smoking status, yes *n* (%)	2 (3.4)	0 (0)	–	–
Drinking status, yes *n* (%)	3 (5.2)	0 (0)	–	–
BES score (M ± SD)	21.60 ± 3.29	5.47 ± 2.22	31.04	0.001
**BIS-11 scores (M** ±**SD)**				
Attentional Impulsiveness	19.50 ± 3.10	16.83 ± 3.16	4.61	0.001
Motor Impulsiveness	22.07 ± 3.63	20.32 ± 3.17	2.77	0.006
Non-planning Impulsiveness	30.24 ± 4.24	28.93 ± 4.03	1.71	0.090
**UPPS-P scores (M** ±**SD)**				
Negative Urgency	32.02 ± 4.17	26.36 ± 4.92	6.72	0.001
Lack of Premeditation	23.76 ± 4.60	23.25 ± 3.99	0.63	0.527
Lack of Perseverance	22.95 ± 3.58	21.24 ± 3.20	2.73	0.007
Sensation Seeking	29.24 ± 6.63	28.64 ± 6.03	0.51	0.611
Positive Urgency	34.34 ± 5.68	29.49 ± 6.18	4.42	0.001
**DDT scores (M** ±**SD)**				
*k-*value/log-transformed	0.03 ± 0.06/−2.07 ± 0.82	0.01 ± 0.02/−2.22 ± 0.62	2.33/1.09	0.023/0.279
**PDT scores (M** ±**SD)**				
**Part A ($20 vs. $80)**				
*h-*value/log-transformed	2.94 ± 3.08/0.23 ± 0.49	3.79 ± 3.71/0.39 ± 0.43	−1.36/−1.92	0.177/0.058
**Part B ($40 vs. $10)**				
*h-*value/log-transformed	2.49 ± 3.33/0.13 ± 0.46	2.09 ± 2.34/0.14 ± 0.38	0.75/−0.21	0.455/0.836
**Part C ($40 vs. $60)**				
*h-*value/log-transformed	2.03 ± 3.25/0.01 ± 0.47	1.86 ± 3.21/−0.03 ± 0.44	0.28/0.43	0.777/0.670

*pBED, probable Binge Eating Disorder; HCs, Healthy Controls; BES, Binge Eating Scale; BIS, Barratt Impulsiveness Scale; UPPS-P, UPPS-P Impulsive Behaviors Scale; DDT, Delay-discounting Test; PDT, Probability Discounting Test; k represents the discounting rate, and h represents the probability discounting rate*.

### Trait Impulsivity

On the BIS-11, the mANOVA models revealed significant between-group differences on Attention Impulsiveness [*F*_(1, 114)_ = 21.061, *p* = 0.001, $\eta_{p}^{2}$ = 0.156] and Motor Impulsiveness [*F*_(1, 114)_ = 8.043, *p* = 0.005, $\eta_{p}^{2}$ = 0.066], but not on Non-Planning Impulsiveness [*F*_(1, 114)_ = 2.971, *p* = 0.087]. *Post-hoc* comparisons found that the pBED group had higher scores on Attentional Impulsiveness (*M*_*d*_ = 2.669, *p* = 0.001, Cohen's *d* = 0.853) and Motor Impulsiveness (*M*_*d*_ = 1.753, *p* = 0.005, Cohen's *d* = 0.514) than the HCs.

On the UPPS-P, the mANOVA models showed significant between-group differences on Negative Urgency [*F*_(1, 114)_ = 44.711, *p* = 0.001, $\eta_{p}^{2}$ = 0.282], Lack of Perseverance [*F*_(1, 114)_ = 7.419, *p* = 0.007, $\eta_{p}^{2}$ = 0.061], and Positive Urgency [*F*_(1, 114)_ = 19.421, *p* = 0.001, $\eta_{p}^{2}$ = 0.146], but not on Lack of Premeditation [*F*_(1, 114)_ = 0.416, *p* = 0.520) or Sensation Seeking (*F*_(1, 114)_ = 0.256, *p* = 0.614]. *Post-hoc* comparisons displayed that pBED group had higher scores on Negative Urgency (*M*_*d*_ = 5.662, *p* = 0.001, Cohen's *d* = 1.241), Lack of Perseverance (*M*_*d*_ = 1.712, *p* = 0.007, *Cohen' s d* = 0.504), and Positive Urgency (*M*_*d*_ = 4.855, *p* = 0.001, *Cohen' s d* = 0.817) than HCs.

### Choice Impulsivity

On the DDT, the mANOVA models displayed no significant between-group differences on the log-transformed *k*-value [*F*_(1, 114)_ = 1.176, *p* = 0.280]. On the PDT, the mANOVA models found no significant between-group differences on the log-transformed *h* values of the Part A [*F*_(1, 114)_ = 3.681, *p* = 0.058], Part B [*F*_(1, 114)_ = 0.044, *p* = 0.835] or Part C [*F*_(1, 114)_ = 0.183, *p* = 0.670].

### Partial Correlation and Linear Regression Outcomes

As seen in Table 2, significant positive correlations were detected between the BES scores and BIS-11 Attentional Impulsiveness, Motor Impulsiveness, Non-planning Impulsiveness, UPPS-P Negative Urgency, Lack of Perseverance, and Positive Urgency scores (*r*_*p*_ = 0.23–0.60, *ps* < 0.05). Nevertheless, no significant correlations were detected between the BES scores and UPPS-P Lack of Premeditation, Sensation Seeking, DDT *k*-value (log-transformed), and PDT *h*-values (log-transformed) of the three parts.

**Table 2:** Partial correlations analyses of impulsivity measures and BES scores (*N* = 117).

**Variables**	**1**	**2**	**3**	**4**	**5**	**6**	**7**	**8**	**9**	**10**	**11**	**12**
1.BES score	–											
2.BIS Attentional Impulsiveness	0.40***	–										
3.BIS Motor Impulsiveness	0.25**	0.50***	–									
4.BIS Non-planning Impulsiveness	0.23*	0.55***	0.58***	–								
5.UPPS-P Negative Urgency	0.60***	0.53***	0.35***	0.28**	–							
6.UPPS-P Lack of Premeditation	0.12	0.31***	0.45***	0.67***	0.11	–						
7.UPPS-P Lack of Perseverance	0.28**	0.53***	0.40***	0.64***	0.31**	0.52***	–					
8.UPPS-P Sensation Seeking	0.08	0.03	−0.12	−0.20*	0.15	−0.17	−0.34***	–				
9.UPPS-P Positive Urgency	0.39***	0.40***	0.32**	0.20*	0.74***	0.03	0.27**	0.18	–			
10.DDT *k* (log-transformed)	0.10	0.12	−0.03	0.01	0.19*	−0.03	0.01	0.10	0.25**	–		
11.PDT Part A *h* (log-transformed)	−0.16	−0.05	0.10	0.10	−0.06	−0.01	0.04	−0.19*	−0.09	−0.18	–	
12.PDT Part B *h* (log-transformed)	−0.08	−0.07	0.11	0.05	−0.01	0.02	0.10	−0.16	0.01	−0.14	0.66***	–
13.PDT Part C *h* (log-transformed)	0.02	0.17	0.18	0.19*	0.11	0.12	0.18	−0.03	0.13	−0.09	0.36***	0.61***

BES, Binge Eating Scale; BIS, Barratt Impulsiveness Scale-11; UPPS-P, UPPS-P Impulsive Behaviors Scale; DDT, Delay-discounting Test; PDT, Probability Discounting Test; k represents the delay discounting rate, and h represents the probability discounting rate. Control variables: BMI, Age, Ethnicity, Gender, Home locality. *p < 0.05, **p < 0.01, ***p < 0.001.

The multivariate linear regression analyses were used to test the effect of BIS-11, UPPS-P, DDT, and PDT scores on BES scores, with a two-step design. BMI was entered in step 1 as the control variable, and the impulsivity scores were entered in step 2 as the predictor variables. Table 3 displayed that only UPPS-P Negative Urgency positively predicted BES scores, after controlling for the effect of BMI [*F*_(13, 103)_ = 7.03, *p* < 0.001; Δ*R*^2^ = 0.37, *p* < 0.001].

**Table 3:** Multivariable linear regression analyses of impulsivity measures on BES scores (*N* = 117).

**Models**	**Standardized coefficient (β)**	***t***	***F***	***R***	***R^**2**^***	***ΔR^**2**^***
**Step 1**			12.96***	0.32	0.10	0.10***
BMI	0.32	3.60***				
**Step 2**			7.03***	0.69	0.47	0.37***
BMI	0.31	4.09***				
BIS Attentional Impulsiveness	0.03	0.24				
BIS Motor Impulsiveness	0.06	0.63				
BIS Non-planning Impulsiveness	0.02	0.15				
UPPS-P Negative Urgency	0.62	5.07***				
UPPS-P Lack of Premeditation	−0.04	−0.39				
UPPS-P Lack of Perseverance	0.09	0.83				
UPPS-P Sensation Seeking	0.00	0.05				
UPPS-P Positive Urgency	−0.14	−1.21				
DDT *k* (log-transformed)	−0.02	−0.25				
PDT Part A *h* (log-transformed)	−0.14	−1.38				
PDT Part B *h* (log-transformed)	0.00	−0.03				
PDT Part C *h* (log-transformed)	−0.01	−0.11				

BES, Binge Eating Scale; BIS, Barratt Impulsiveness Scale-11; UPPS-P, UPPS-P Impulsive Behaviors Scale; DDT, Delay-Discounting Test; PDT, Probability Discounting Test; k represents the delay discounting rate, and h represents the probability discounting rate. Control variable: BMI. Dependent variable: BES scores. ***p < 0.001.

### Logistic Regression Outcomes

The binary logistic regression models were conducted to examine the effects of the impulsivity scores on binge-eating behavior. A two-step design was used: BMI was entered in step 1 as the control variable, and the three dimensions of BIS-11 (Attentional Impulsiveness, Motor Impulsiveness, and Non-planning Impulsiveness), five dimensions of UPPS-P (Negative Urgency, Lack of Premeditation, Lack of Perseverance, Sensation Seeking, and Positive Urgency), DDT *k*-value (log-transformed), and PDT *h* values (log-transformed) were entered in step 2. Table 4 revealed that only Negative Urgency positively predicted binge eating behavior (*OR* = 1.54, *p* < 0.001, *NagelkerkeR*^2^ = 0.650 for the model).

**Table 4:** Logistic regression analyses of impulsivity scores on binge eating controlling for BMI (*N* = 117).

**Models**	**Probable BED group vs. healthy controls**		
	***B***	**Wald χ^2^**	**OR (95% CI)**
**Step 1**			
BMI	0.57	12.99***	1.77 (1.30–2.42)
**Step 2**			
BIS Attentional Impulsiveness	0.05	0.13	1.05 (0.81–1.35)
BIS Motor Impulsiveness	0.12	1.13	1.13 (0.90–1.41)
BIS Non-planning Impulsiveness	0.01	0.00	1.01 (0.80–1.28)
UPPS-P Negative Urgency	0.43	14.59***	1.54 (1.24–1.93)
UPPS-P Lack of Premeditation	−0.08	0.62	0.92 (0.76–1.13)
UPPS-P Lack of Perseverance	0.09	0.47	1.09 (0.85–1.41)
UPPS-P Sensation Seeking	−0.01	0.06	0.99 (0.88–1.10)
UPPS-P Positive Urgency	−0.07	0.85	0.94 (0.81–1.08)
DDT *k* (log-transformed)	−0.19	0.21	0.83 (0.37–1.86)
PDT Part A *h* (log-transformed)	−1.24	2.50	0.29 (0.06–1.35)
PDT Part B *h* (log-transformed)	0.02	0.00	1.02 (0.13–8.14)
PDT Part C *h* (log-transformed)	−0.50	0.40	0.61 (0.13–2.87)

BED, Binge Eating Disorder; BIS, Barratt Impulsiveness Scale-11; UPPS-P, UPPS-P Impulsive Behaviors Scale; DDT, Delay-discounting Test; PDT, Probability Discounting Test; k represents the delay discounting rate, and h represents the probability discounting rate. CI, confidence interval; OR, odds ratio. ***p < 0.001.

## Discussion

To the best of our knowledge, the present study was the first to examine the associations between trait impulsivity, choice impulsivity, and binge-eating behavior in non-clinical samples. The results supported our hypotheses that individuals with probable binge eating disorder (pBED) might have elevated impulsive personality traits than the healthy controls. Specifically, the probable BED subjects showed higher levels of trait impulsivity on the BIS-11 (i.e., Attentional Impulsiveness, Motor Impulsiveness) and UPPS-P (i.e., Negative Urgency, Lack of Perseverance, Positive Urgency). However, the probable BED group had a normal level of choice impulsivity both on the DDT and the PDT, compared with the healthy controls. Significant positive correlations were found between BES scores and most trait impulsivity scores, including BIS-11 Attentional Impulsiveness, Motor Impulsiveness, Non-planning Impulsiveness, UPPS-P Negative Urgency, Lack of Perseverance, and Positive Urgency. More importantly, regression models showed that only Negative Urgency positively predicted binge eating behavior as a potential risk factor. These findings suggested that different impulsivity facets were separately associated with BED, and certain trait impulsivity (Negative Urgency) might be considered a hallmark for BED in non-clinical young adults.

Increased impulsivity has been proposed as a phenotype for addictive disorders as well as within the clinical obesity spectrum, and it might also increase the onset of BED (12). However, few studies have focused on the relationship between impulsivity and binge eating in non-treatment-seeking individuals with normal weight. The current study investigated the associations of trait impulsivity, choice impulsivity, and binge-eating behavior in common populations (i.e., young adult college students). The data showed that individuals with probable BED had elevated scores on measurements of trait impulsivity (i.e., Attentional Impulsiveness, Motor Impulsiveness, Negative Urgency, Lack of Perseverance, and Positive Urgency), consistent with previous reports on BED (54–57) and addictive disorders (58, 59).

Furthermore, positive correlations were detected between the BES scores and these impulsivity scores (Table 2). However, only Negative Urgency displayed the main effect as a significant indicator for binge-eating behavior in the regression models (Tables 3, 4). These findings suggested that elevated Negative Urgency might represent a preclinical susceptibility marker for binge eating disorder, although longitudinal studies are needed to clarify whether Negative Urgency precedes the onset of binge eating behavior or as a consequence of BED. Nevertheless, our first direct evidence in the non-treatment-seeking populations showed that specific trait of impulsivity (i.e., Negative Urgency) was overtly enhanced in binge-eating behavior (60–62). Negative Urgency reflects a typical tendency to act impulsively under the condition of extreme negative emotions (63). Individuals with elevated Negative Urgency seemed more likely to be involved into binge eating in order to deal with negative emotions, and as a result, their binge-eating behaviors would be further reinforced or deteriorated (64). Our results increased new knowledge to the current literature that Negative Urgency could play a key role for binge eating even in the non-clinical samples, as a possible susceptible hallmark of binge-eating behavior, which should promote a better understanding of the pathogenesis of BED. More interestingly, the proposed “Emotional Regulation Model” of BED emphasized that negative emotions might serve as a trigger component for binge eating, and impulsivity could possibly offer one explanation for the cause of binge eating triggered by negative emotions (65). In this respect, Negative Urgency might potentially represent a characteristic trait of impulsivity linking negative emotions to binge eating behavior that individuals with BED might react to binge eating with dysfunctional emotion regulation strategy due to their high level of impulsivity (65). If this is the case, it would be of help to further develop innovative therapeutic approaches for BED. Indeed, a recent study found that food-related impulsivity measured by laboratory tasks could be reduced by cognitive behavioral interventions and suggested that such clinical treatments might also be effective in modifying trait impulsivity associated with negative emotional states (e.g., Negative Urgency) in patients with BED (66). Nevertheless, further evidence-based experiments and clinical trials are warranted to verify the possible role of Negative Urgency both in the onset and development of binge eating behavior and in the treatment outcomes among different samples of binge eating disorder.

On the other side, the probable BED group did not show an aberrant pattern of choice impulsivity. The data revealed that individuals with probable BED performed similarly with the healthy controls on the Delay Discounting Test (DDT) and the Probability Discounting Test (PDT). Moreover, the DDT *k* value and PDT *h*-values were not significantly associated with or predictive of binge eating (Tables 2–4). Previous studies found that obese women with BED had higher discounting degrees of delayed rewards (67), and addictive drug abusers displayed a lower risk aversion compared to matched controls (68, 69). Among clinical samples of BED as well as those of obesity without BED, reduced reward processing in the striatal and amygdala regions indicated motivational hypofunction to non-food rewards (70, 71). Nonetheless, a longitudinal study showed that the ventromedial prefrontal cortex (vmPFC) activation did not display a significant effect on the severity of binge-eating behaviors in adolescent girls (72). Therefore, more parallel studies should be conducted to investigate the processes of delay gratification and risk aversion in both clinical and non-clinical samples of BED in the future. Meanwhile, a notable question related to choice impulsivity in BED should be taken into consideration that these two tasks (i.e., DDT and PDT) mainly contain hypothetical monetary rewards as the stimulus, which might reduce the suitability and validity of these tasks used in binge eating (37). Previous studies using non-monetary rewards on the DDT found evidence of increased choice impulsivity in BED individuals, with the largest effect sizes observed for food rewards (41). Thus, focus on food-related impulsivity should be paid more attention to in the investigation and might be conducive to better understanding the pathology of BED (12, 73).

Several limitations should be noted in the current study. Firstly, this study was a cross-sectional design in nature, and thus could not draw a causal conclusion between these impulsivity aspects and BED. Moreover, the samples mainly consisted of young adult college students and the results could not be generalized to clinical samples with serious binge-eating problems. Future research should investigate the relationship of specific trait impulsivity (e.g., Negative Urgency) with binge-eating behaviors in more severe clinical patients with BED. Thirdly, given that our study mostly focused on some limited aspects of impulsivity (i.e., trait impulsivity and choice impulsivity) measured by self-report scales, these findings should be interpreted more carefully because of the possible subjective bias, and other important facets of impulsivity (e.g., inhibitory control) should be investigated using more objective tasks. Especially, impulsivity is a quite complex and multidimensional theoretical concept (9), with diverse measurements probably evaluating differentiated facets of impulsivity. In our study, the BIS-11 and UPPS-P used are basically self-report questionnaires about different types of trait impulsivity that may reflect enduring impulsivity constructs, while the DDT and PDT are monetary reward-based decision-making tasks assessing behavioral choice impulsivity that may measure rather the current state of impulsive behaviors (e.g., delay discounting) (19, 74). As a result, the very low correlations between trait impulsivity (BIS-11, UPPS-P) and choice impulsivity (DDT, PDT) scores in this study (Table 2) further indicated that these two types of impulsivity could be largely uncorrelated and represent distinct pathways and processes of impulsivity (74). Therefore, more intensive studies are needed to further uncover the underlying mechanisms of these impulsivity categories connected to binge eating and other disorders. In addition to these points, another potential limitation should also be noted in the current study that the classification of probable BED was based on the BES scores without standard clinical diagnoses for BED. Thus, these participants were in fact undiagnosed non-clinical samples, although they might experience binge-eating behaviors and problems in the routine daily life. In the meantime, possible compensatory behaviors that could be associated with binge eating were not evaluated and excluded in the subjects, considering certain conditions such as life stress and negative emotions (e.g., depression, anxiety) are important risk factors for BED (75). Future similar studies should consider more standard diagnoses and compensatory behaviors.

Despite these limitations, the present study firstly looked into the associations between various aspects of impulsivity and binge-eating behavior in non-clinical samples of probable BED with a case-control design. Our results indicated that Attentional Impulsiveness, Motor Impulsiveness, Negative Urgency, Lack of Perseverance, and Positive Urgency were elevated in probable BED, and in particular, Negative Urgency was the only positive predictor of binge eating behavior. These findings suggested that typical facets of trait impulsivity, which have been recognized in addictive disorders, were associated with binge eating in young adults, while choice impulsivity was not aberrantly seen in the same probable BED sample.

## Data Availability Statement

The raw data supporting the conclusions of this article will be made available by the authors, without undue reservation.

## Ethics Statement

The studies involving human participants were reviewed and approved by the Human Research Ethics Committee at the Guizhou Medical University. The patients/participants provided their written informed consent to participate in this study.

## Author Contributions

W-SY designed the study, wrote the protocols, directed the study, and wrote the first draft of the manuscript. D-HZ performed the main data analysis and assisted in writing the first draft of the manuscript. M-ML contributed to the assessments and data collection. All authors contributed to this article and have approved the final manuscript.

## Funding

This study was supported by the National Natural Science Foundation of China (Nos: 32060195 and 31560284) to W-SY.

## Conflict of Interest

The authors declare that the research was conducted in the absence of any commercial or financial relationships that could be construed as a potential conflict of interest.

## Publisher's Note

All claims expressed in this article are solely those of the authors and do not necessarily represent those of their affiliated organizations, or those of the publisher, the editors and the reviewers. Any product that may be evaluated in this article, or claim that may be made by its manufacturer, is not guaranteed or endorsed by the publisher.
